# Identification and validation of biomarkers related to ferroptosis in idiopathic pulmonary fibrosis

**DOI:** 10.1038/s41598-025-93217-9

**Published:** 2025-03-13

**Authors:** Ming Yue, Rumei Luan, Dongyan Ding, Yuhong Wang, Qianfei Xue, Junling Yang

**Affiliations:** 1 Department of Respiratory Medicine, The Second Hospital of Jilin University, Changchun, China; 2 Department of Respiratory Medicine, Shandong First Medical University Affiliated Provincial Hospital, Jinan, China; 3 Department of Respiratory Medicine, The 958 Hospital of Chinese PLA/Jiangbei Campus, The First Affiliated Hospital of Army Medical University, Chongqing, China; 4 Department of Respiratory Medicine, Jilin Central General Hospital, Jilin, China; 5 Hospital of Jilin University, Changchun, China

**Keywords:** Idiopathic pulmonary fibrosis, Ferroptosis, ATM, Immune cell infiltration, Data acquisition, Data mining, Data processing, Databases, Biomarkers, Diagnostic markers, Respiratory tract diseases

## Abstract

Idiopathic pulmonary fibrosis (IPF) is a kind of interstitial lung disease (ILD). It has a high incidence rate and mortality. Its pathogenesis remains unclear. So far, no effective methods have been found for the early diagnosis of IPF. Ferroptosis has been reported to be critical in the initiation and progression of IPF. Therefore, our aim was to identify the hub gene related to ferroptosis co-expressed in the peripheral blood and pulmonary tissue of patients with IPF. Sequencing data were obtained from the Gene Expression Omnibus database. A comprehensive analysis was conducted on the differentially expressed genes (DEGs) to extract ferroptosis-related differentially expressed genes (FRDEGs). The results showed that ferroptosis-related signal paths were highly enriched in IPF, and 10 FRDEGs were identified.The hub gene was predicted through protein-protein interactions (PPI) and Cytoscape. The diagnostic utility of the hub gene was proven by enzyme-linked immunosorbent assay (ELISA) in serum and by immunohistochemistry (IHC) in pulmonary tissues. The results of ELISA indicated that the levels of ATM in the serum of patients with IPF were significantly lower than the normal levels. In contrast, the results of IHC showed that the expression of ATM in the pulmonary tissues of IPF patients exhibited a notably elevated trend. The immune status was assessed by the CIBERSORT method and so was the relevance between ATM and immune cells. These findings unveiled significant differences in various immune cell types in peripheral blood and pulmonary tissue between the IPF group and the control group. Furthermore, ATM was associated with various immune cells. This study suggests that as a ferroptosis-related gene, ATM assumes a pivotal role in the diagnosis and treatment of IPF. This discovery presents a novel approach for the clinical diagnosis and therapy of IPF.

## Introduction

Idiopathic pulmonary fibrosis (IPF) is a kind of interstitial lung disease (ILD) defined by chronic and evolving causes of unknown origin^1^. The destruction of pulmonary tissue results from the over-proliferation of fibroblasts, the accumulation of extracellular matrix (ECM) and the epithelial-mesenchymal transition (EMT)^2,3^. Recently, the prevalence and death rates of IPF have been increasing, and the average survival time after diagnosis is approximately 3–5 years^4^. The pathogenesis of IPF is unclear, and the treatment of IPF is currently limited to oral nintedanib, pirfenidone, or lung transplantation^1^.

Ferroptosis is a recently discovered cell death mechanism, which is distinct from apoptosis and autophagy. The characteristic of ferroptosis is the accumulation of iron dependent lipid peroxides to lethal levels^5^. Ferroptosis is closely linked to the onset and development of many diseases^6^. Pulmonary fibrosis (PF) is closely associated with ferroptosis. Bleomycin can induce ferroptosis in pulmonary epithelial cells, and the ferroptosis inhibitor liproxstatin-1 and the iron chelator deferoxamine can reduce the severity of bleomycin-induced PF^7^. Particulate matter 2.5 from diesel exhaust fumes significantly worsens the degree of bleomycin-induced PF through ferroptosis^8^. Massive iron accumulation and ferroptosis of alveolar type II (ATII) cells in the pulmonary tissues of patients with PF indicate that iron-induced ferroptosis worsens PF^9^. In summary, it is essential to study the ferroptosis genes associated with IPF, which are likely to be potential diagnostic markers and targets for the treatment of IPF.

Recently, more studies have indicated that immune cells play a significant part in IPF, and ferroptosis worsens the severity of IPF by affecting immune cells. In the bronchoalveolar lavage fluid of patients with IPF, the increasing number of iron-containing macrophages causes macrophages to release reactive oxygen species (ROS). This promotes lipid peroxidation in IPF, indicating that iron accumulation in macrophages may worsen IPF^10^. An increase in monocyte number heightens the risk of IPF development, hospitalization and death^11^. The matrix metalloproteinase-19 (MMP19) promotes EMT, migration, and permeability of human pulmonary microvascular endothelial cells in vitro. In vivo, MMP19 stimulates monocyte infiltration into the alveoli, thereby worsening bleomycin-induced PF^12^. Regulatory T cells (Tregs) are essential for regulating immunity and sustaining immunological tolerance. However, the effect of Tregs on IPF has shown conflicting outcomes. Kotsianidis et al. found that the number of Tregs decreased in the peripheral blood and bronchoalveolar lavage of patients with IPF^13^, which contradicts the findings of Galati et al.^14^. Although some studies have shown that Tregs can release IL-10 to prevent TGF-β-induced fibrosis^15^, Tregs aggravate TGF-β-induced collagen deposition in the lungs of mice^16^. Additionally, interferon gamma (IFN-γ) produced by T cells has been found to be inhibited by Tregs^17^. Th1 cells have anti-fibrotic effect by producing IFN-γ and interleukin(IL)-12, whereas Th2 cells stimulate myofibroblasts by producing cytokines, leading to the progression of IPF^18^. Regulatory B cells (Bregs) inhibit T cell-driven immune responses, and produce IL-10 and TGF-β with anti-inflammatory effects^19^. Neutrophils produce elastase (NE), MMP, and their antiprotease (TIMP). NE promotes inflammation by activating TGF-β, thereby exacerbating IPF^20^. The balance between MMP and TIMP plays a major part in the accumulation and reduction of ECM in PF^21^. Macrophages release various cytokines during wound healing, regulating fibroblast activation and ECM accumulation, and thus promoting scar formation. Macrophages also participate in inhibiting fibrosis by secreting tissue inhibitors of MMP^22^. M1 macrophages participate in the early inflammatory phase of PF, whereas M2 macrophages are involved in resolving inflammation in the later phase of PF^23^. These studies collectively demonstrate the intricate role of various immune cells in the onset and progression of IPF.

Research on ferroptosis-related genes (FRGs) in bronchoalveolar lavage fluid and pulmonary tissue has been reported^10^, but no studies have validated them using human samples. Moreover, if other animal samples are used instead of human samples for validation, the authenticity of the experimental results may be greatly reduced. Thus, we aimed to fill this gap by validating the ferroptosis - related gene using human samples. Therefore, we hypothesize that distinct FRGs are co-expressed in human peripheral blood and pulmonary tissue, and the change in ferroptosis levels may be related to immune cell infiltration.

In this study, bioinformatics analysis methods were comprehensively employed to precisely screen out the co-expressed hub ferroptosis-related gene in the pulmonary tissues and peripheral blood of patients with IPF. Subsequently, we conducted validation of this gene using human serum samples via the ELISA method and human pulmonary tissue samples through the IHC method. This study also revealed the inherent association between the gene and the immune infiltration phenomenon in IPF. From the perspective of ferroptosis, it put forward new insights into the clinical diagnosis and treatment of IPF, opening up a new perspective for the treatment of ferroptosis in IPF.

## Materials and methods

### GEO

**Fig. 1:**
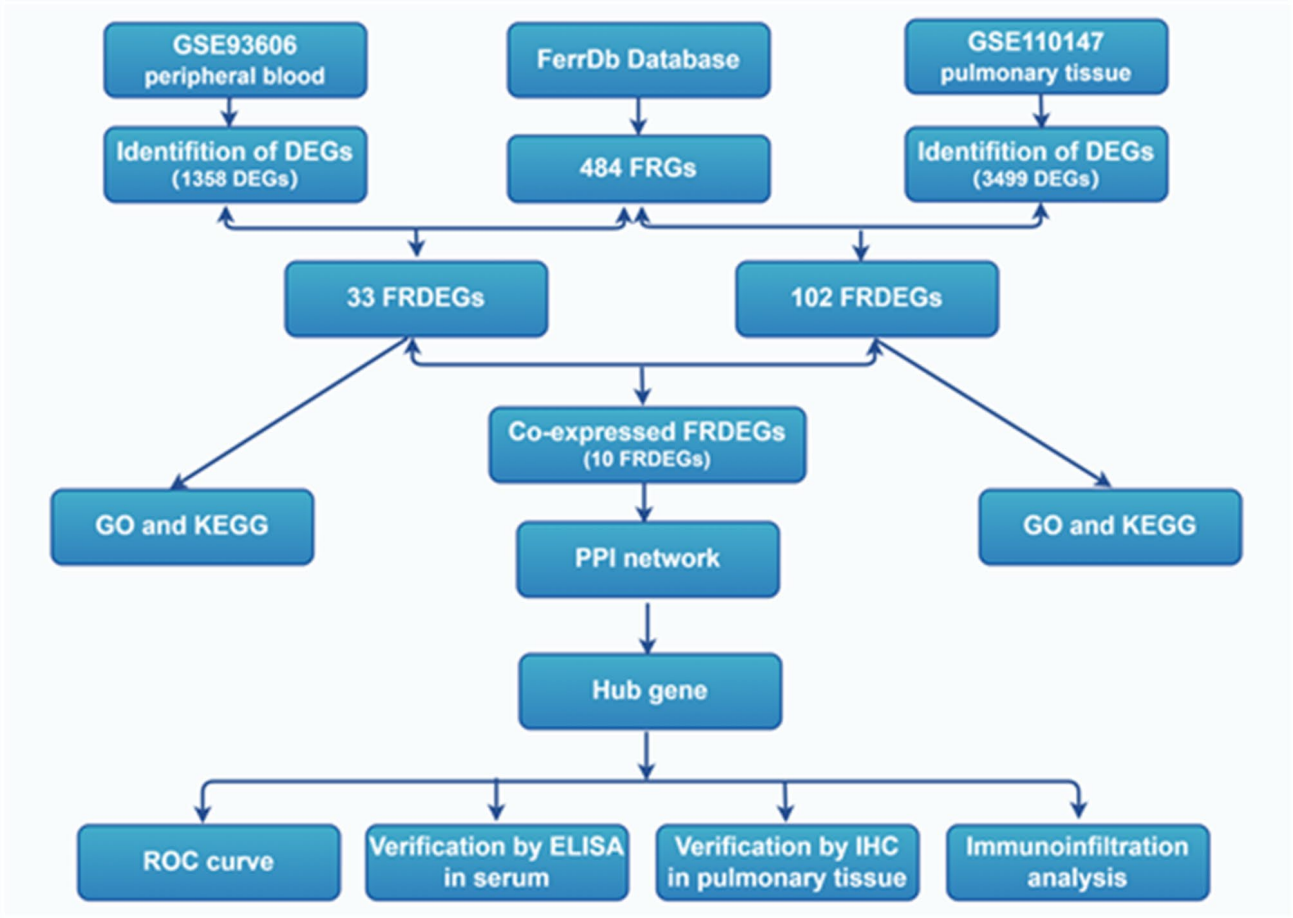
Workflow diagram of this study. This workflow outlines the process starting with data preprocessing, then using bioinformatics to identify the hub gene, followed by validating it in serum through ELISA and in pulmonary tissues through IHC, and culminating in the analysis of immune cell infiltration.

The datasets GSE93606 and GSE110147 from the Gene Expression Omnibus (GEO) database (https://www.ncbi.nlm.nih.gov/geo/*)* were selected. GSE93606 (on the GPL11532 platform) is a dataset on peripheral blood from IPF and controls. GSE110147 (on the GPL6244 platform) is a dataset on pulmonary tissue from IPF and controls. The workflow diagram of this study is shown in Fig. 1.

### Differential expression analysis

DEGs of GSE110147 and GSE93606 were identified using the limma package. Expression values were log2-transformed and normalized by quantile normalization. Age and smoking status were included as covariates in the design matrix to minimize potential confounding effects. For GSE110147, DEGs were identified using the thresholds of |log2FC| > 2 and FDR < 0.05. For GSE93606, the criteria were set as |log2FC| > 0.31152 and FDR < 0.05. The Benjamini-Hochberg procedure was applied to control the false discovery rate for multiple testing correction. Volcano plots were generated using the ggplot2 package to visualize the distribution of DEGs, where genes were categorized as up-regulated, down-regulated, or not significant based on the aforementioned criteria.

### DEGs associated with ferroptosis

The last 484 FRGs obtained after removing duplicated genes were searched from the FerrDb database (http://www.zhounan.org/ferrdb)^24^. GSE93606, GSE110147, and 484 FRGs were simultaneously used to identify intersections (https://bioinfogp.cnb.csic.es/tools/venny/), which were shown as Venny plots, to acquire FRDEGs.

### Analysis of FRDEGs using GO and KEGG pathways

GO and KEGG^25–27^ pathway enrichment analyses were presented using the Metascape online tool (https://metascape.org/gp/index.html^28^ and https://bioinformatics.com.cn/*).*

### PPI analysis and correlation analyses of FRDEGs

The STRING online database (https://string-db.org/) and Cytoscape software (version 3.9.1) were used to analyze the interactions between FRDEGs^29^. Set cutoff values in the STRING online database (interaction score > 0.40). The STRING results were then loaded into the Cytoscape software. Finally, the highest-scoring gene in the subnetworks was selected as hub gene.

### Study population

This retrospective cohort study enrolled 95 patients with IPF and 60 healthy participants from the Second Hospital of Jilin University, Jilin Provincial People’s Hospital and Jilin Central Hospital from March 2022 to March 2024. Data on sex, age, laboratory results, and pulmonary function test results were collected. Venous blood samples (3 ml) were collected from each participant. Pulmonary tissue samples were obtained from three patients with IPF and three controls (the controls were normal pulmonary tissue adjacent to lung cancer). Eligibility criteria were used as follows: 1). patients were diagnosed with IPF according to ATS/ERS/JRS/ALAT IPF guidelines^30^; 2). patients must have undergone high-resolution computed tomography (HRCT) examination assessed by two radiologists independent of clinical knowledge. Exclusion criteria were used as follows: 1). other factors contributing to interstitial lung disease (ILD), such as connective tissue diseases, household or occupational exposure, and drug toxicity; 2). factors affecting serum levels of patients, including liver failure, kidney failure, cardiac insufficiency, inflammation, tumor, and lung transplantation. This study was approved by the Medical Ethics Committee of the Second Hospital of Jilin University (approval no. 2024161).

### ELISA determination

The collected peripheral blood samples were centrifuged at 3000 × g for 10 min, and the supernatants were collected and reserved at − 80 ℃. Commercial ELISA kits (Shanghai Enzyme-Linked Biotechnology Co., Ltd., Shanghai, China) were used to determine the concentrations of serum-related indicators according to the reagent instructions^31^. An enzyme marker instrument (Thermo Scientific, USA) at a wavelength of 450 nm measured the absorbance of each well, and the concentration of each sample was calculated using the standard curve.

### IHC

Details of the IHC staining procedure was previously described^32^. After sealing, the slides were examined and captured under a microscope (Olympus, Japan). ATM is primarily expressed in the cell nucleus^33^. The ImageJ program was applied to measure the percentage of positive cells among the total cells. The following primary antibody was used: ATM (1:200 dilution; MedChemExpress, China).

### Immune cell infiltration

CIBERSORT was a deconvolution-based algorithm, which analyzed the expression matrix of 22 human immune cell subtypes using the linear support vector regression principle^34^. It was employed to explore differences in immune cells between patients with IPF and controls. Additionally, the relevance between the ferroptosis hub gene and immune cells was analyzed.

### Statistical analysis

The statistical analysis of differential Expression was performed using R software (version 4.1.0). SPSS software (version 20.0, Chicago, IL, USA) and GraphPad Prism software (version 5.0, San Diego, CA, USA) were used for statistical analysis. Categorical variables were described as counts (percentages), and variations between groups were analyzed by the chi-square test. Normally distributed variates were represented by mean ± standard deviation (x ± SD), and variations between groups were assessed by the t-test. Non-normally distributed variates were represented as median and interquartile range, and the wilcoxon rank-sum test was used to contrast differences between groups. Spearman’s correlation coefficient was employed for assessing the correlation between variates. The predictive value of related variables for disease diagnosis was analyzed by the area under the curve (AUC) of the receiver operating characteristic (ROC) curve. It was significant statistically when the value of P was less than 0.05 (*p < 0.05, **p < 0.01, ***p < 0.001, ****p < 0.0001).

## Results

### DEGs in the peripheral blood and pulmonary tissues were collected

**Fig. 2:**
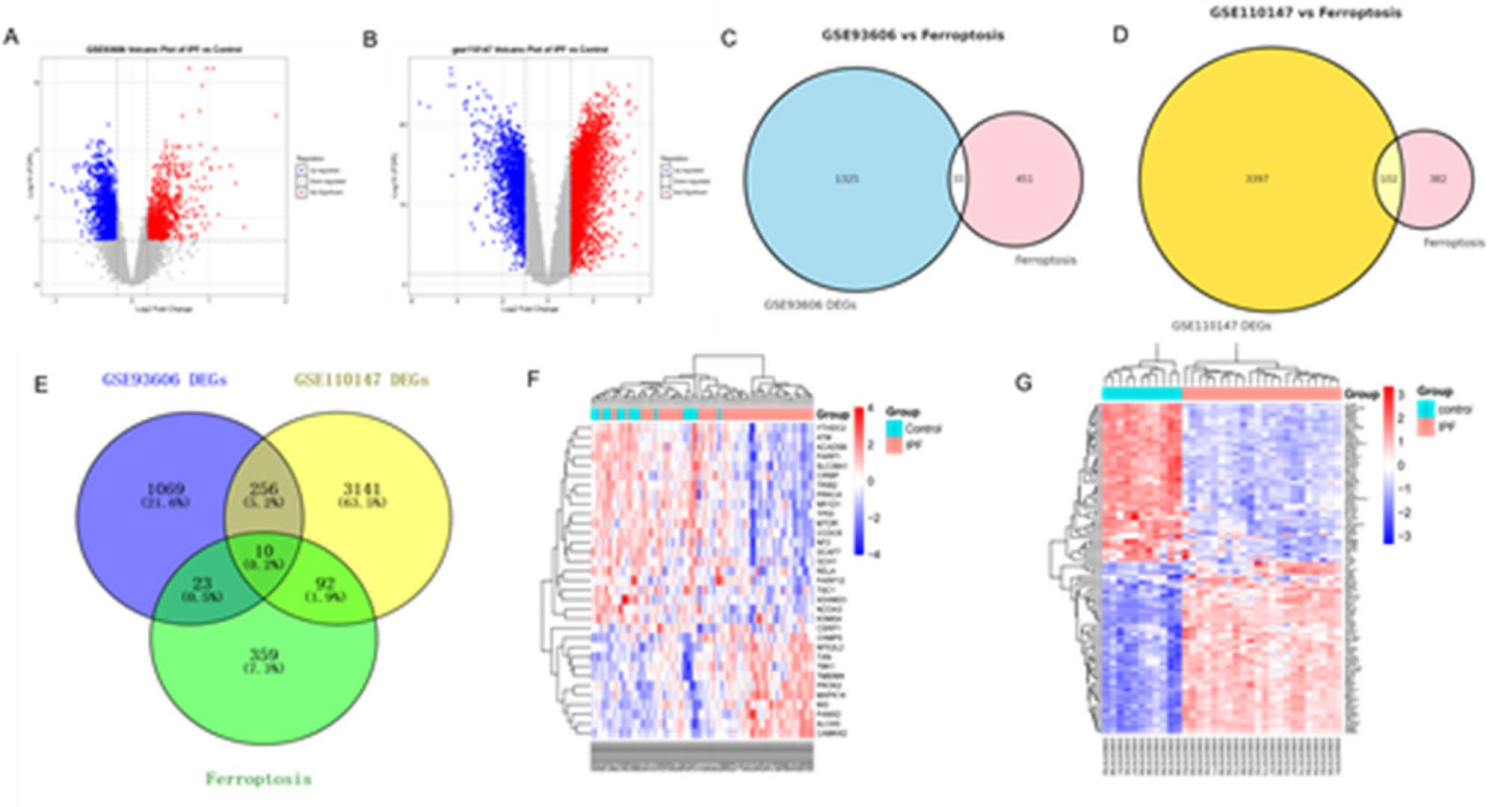
(**A**) Volcano plot of DEGs in GSE93606 |log2FC| > 0.31152; (**B**) Volcano plot of DEGs in GSE110147 |log2FC| > 2; (**C**) Identification of 33 overlapping genes between the DEGs of GSE93606 and FRGs; (**D**) Identification of 102 overlapping genes between the DEGs of GSE110147 and FRGs; (**E**) The DEGs in GSE93606 and GSE110147 intersected with FRGs; (**F**) Heat map of 33 FRDEGS in GSE93606; (**G**) Heat map of 102 FRDEGS in GSE110147.

Analysis of the GSE93606 dataset revealed DEGs in peripheral blood samples from patients with IPF compared to healthy controls (HC). Using the criteria of |log2FC| > 0.31152 and FDR < 0.05, 1358 DEGs were identified, with their distribution visualised in the volcano plot (Fig. 2A). In the pulmonary tissue dataset (GSE110147), more stringent criteria (|log2FC| > 2 and FDR < 0.05) were applied and 3499 DEGs were identified. The distribution of these DEGs was illustrated in the volcano plot (Fig. 2B). To identify FRDEGs, we intersected the DEGs from each dataset with known FRGs. This analysis revealed 33 FRDEGs in the GSE93606 dataset and 102 FRDEGs in the GSE110147 dataset, as depicted in the Venn diagrams (Fig. 2C and D) and the expression patterns of these FRDEGs were visualised in heatmaps (Fig. 2F and G, respectively). Further analysis identified 10 FRDEGs common to both datasets (Fig. 2E): ACADSB, ALOX5, ATM, KDM5A, NFE2L2, PARP1, PARP12, RELA, TBK1, and YTHDC2.

### Analysis of FRDEGs and their enrichment in the datasets

**Fig. 3:**
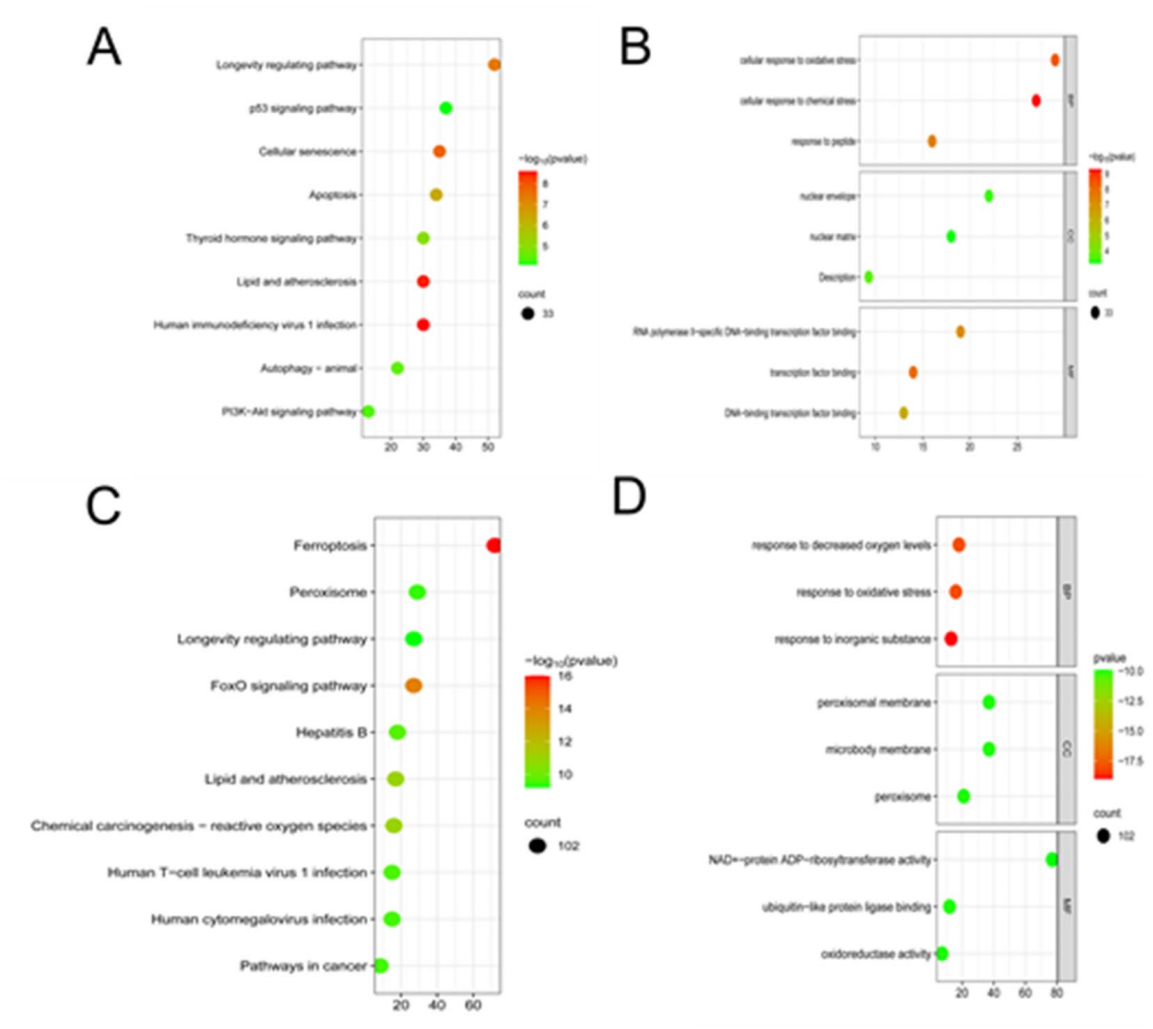
GO and KEGG analysis: (**A**) KEGG analysis of 33 FRDEGs in GSE93606; (**B**) GO enrichment analysis of 33 FRDEGs in GSE93606; (**C**) KEGG analysis of the 102 FRDEGs in the GSE110147 dataset; (**D**) GO enrichment analysis of 102 FRDEGs in the GSE110147 dataset.

The KEGG, MF, BP, and CC analyses of 33 FRDEGs in GSE93606 were obtained using Metascape (Fig. 3A and B). The KEGG pathways mainly included the longevity regulation pathway, P53 signaling pathway, cellular senescence, and apoptosis. MF enrichment analysis involved RNA polymerase II-specific DNA-binding transcription factor binding, transcription factor binding and DNA-binding transcription factor binding. BP enrichment analysis mostly concentrated on cellular response to oxidative stress. CC enrichment analysis was predominantly related to the nuclear envelope.

The KEGG, MF, BP, and CC analyses of 102 FRDEGs in GSE110147 were obtained using Metascape (Fig. 3C and D). The KEGG pathways included ferroptosis, FoxO signaling pathway, lipid and atherosclerosis, and chemical carcinogenesis-reactive oxygen species (ROS). MF enrichment analysis revealed oxidoreductase activity, ubiquitin-like protein ligase binding, and NAD+-protein ADP-ribosyltransferase activity. CC enrichment analysis also revealed peroxisomal membrane, microbody membrane, and peroxisome.

### PPI network analysis

**Fig. 4:**
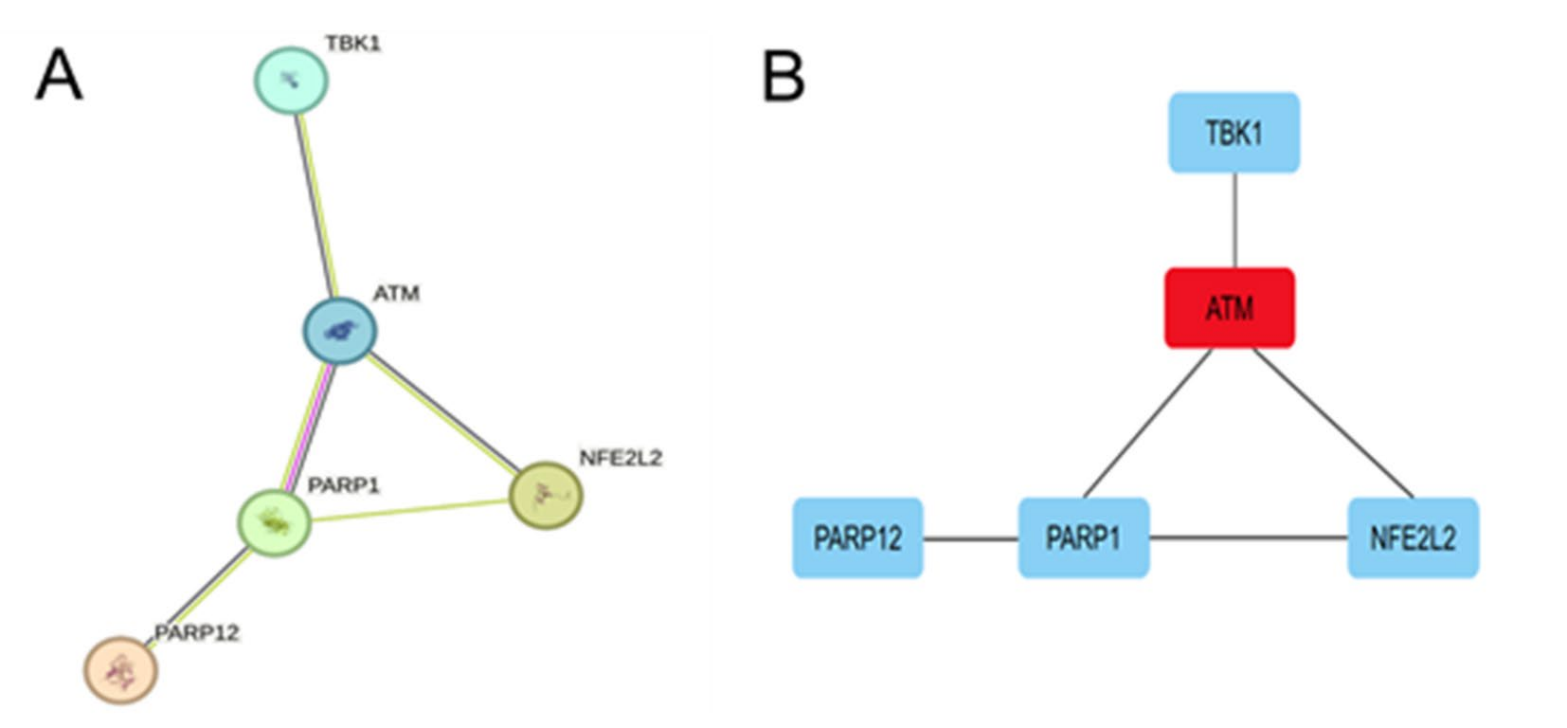
Protein-protein interaction(PPI) network of ferroptosis DEGs: (**A**) PPI maps of 10 FEDEGs; (**B**) Subnetwork of hub gene from the PPI network.

The 10 FRDEGs were analysed online using STRING, and the network was arranged to the default cut-off value (interaction score > 0.4) (Fig. 4A). The hub gene was figured out by the Cytoscape software. The gene with the highest score (ATM) was selected as the hub gene (Fig. 4B).

### ATM expression in the datasets

**Fig. 5:**
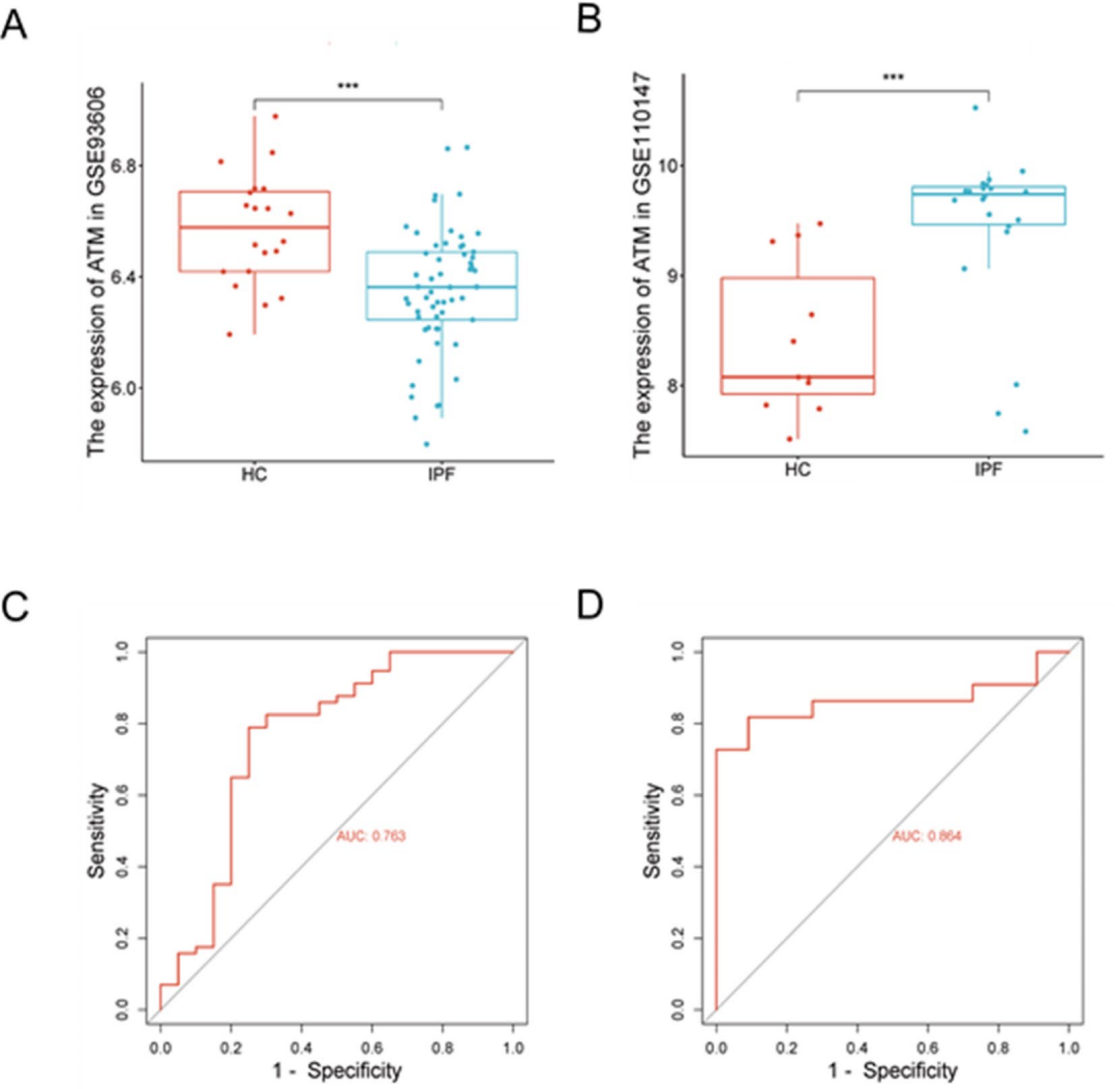
Use of ATM to diagnose IPF in datasets: (**A**) ATM concentration in the serum of patients with IPF and HC in the dataset GSE93606; (**B**) Expression of ATM in the pulmonary tissue of patients with IPF or controls in the dataset GSE110147; (**C**) ROC curve of serum ATM gene for diagnosis of IPF in the dataset GSE93606; (**D**) ROC curve of pulmonary tissue ATM for diagnosis of IPF in dataset GSE110147. ***p < 0.001, ****p < 0.0001.

In the GSE93606 dataset, the ATM concentrations in the serum of patients with IPF were significantly downregulated in contrast to those of HC (Fig. 5A), and the AUC was 0.763 (Fig. 5C). Conversely, in the GSE110147 dataset, ATM expression in the pulmonary tissue of patients with IPF were significantly upregulated (Fig. 5B), and the AUC was 0.864 (Fig. 5D).

### Baseline features of the study participants

Age, sex, and body mass index did not significantly differ between patients with IPF and HC. According to baseline results from their pulmonary function tests, patients with IPF had medium lung damage, who had a mean predicted FVC% of 72.84 ± 9.49 and a mean predicted D_LCO_% of 49.13 ± 8.88 (Table 1).

**Table 1:** Baseline features of the study participants.

	Healthy controls (*n* = 60)	IPF (*n* = 95)	Statistics	*P*-value
Male, n (%)	29 (48.33)	46 (48.40)	*χ*^*2*^ = 0.000	0.992
Age (years)	69.72 ± 8.563	69.07 ± 7.669	t = 0.486	0.628
BMI (kg/m^2^)	22.92 (22.12, 23.41)	22.59 (20.24, 23.50)	z = 1.699	0.089
Smoking status				
Never smokers, n (%)	48 (80.0)	65 (68.4)	*χ*^*2*^ = 2.496	0.114
Ex-smokers, n (%)	6 (10.0)	17 (17.9)	*χ*^*2*^ = 1.814	0.178
Current mokers, n (%)	6 (10.0)	13 (13.7)	*χ*^*2*^ = 0.464	0.496
WBC (*10^9/L)	7.04 ± 2.13	7.30 ± 2.37	t = 0.680	0.497
CRP (mg/dL)	0.48 (0.35, 0.59)	0.43 (0.34, 0.54)	z = 1.566	0.117
PCT (ng/mL)	0.20 (0.11, 0.24)	0.17 (0.09, 0.24)	z = 0.636	0.525
ATM (pg/mL)	3012.62 (2226.87, 3568.81)	1182.88 (820.56, 1864.19)	z = 8.005	< 0.0001
FVC (% of predicted)	N/A	72.84 ± 9.49		
D_LCO_ (% of predicted)	N/A	49.13 ± 8.88		
FEV_1_ (% of predicted)	NA	70.38 ± 10.71		

*IPF* idiopathic pulmonary fibrosis, *BMI* body mass index, *WBC* white blood cell, *CRP* C-reactive protein, *PCT* procalcitonin, *ATM* ataxia telangiectasia mutated, *FVC* forced vital capacity, *D*_*LCO*_ diffusion capacity for carbon monoxide, *FEV*_*1*_ forced expiratory volume in one second.

### Verification of serum ATM concentrations by ELISA

**Fig. 6:**
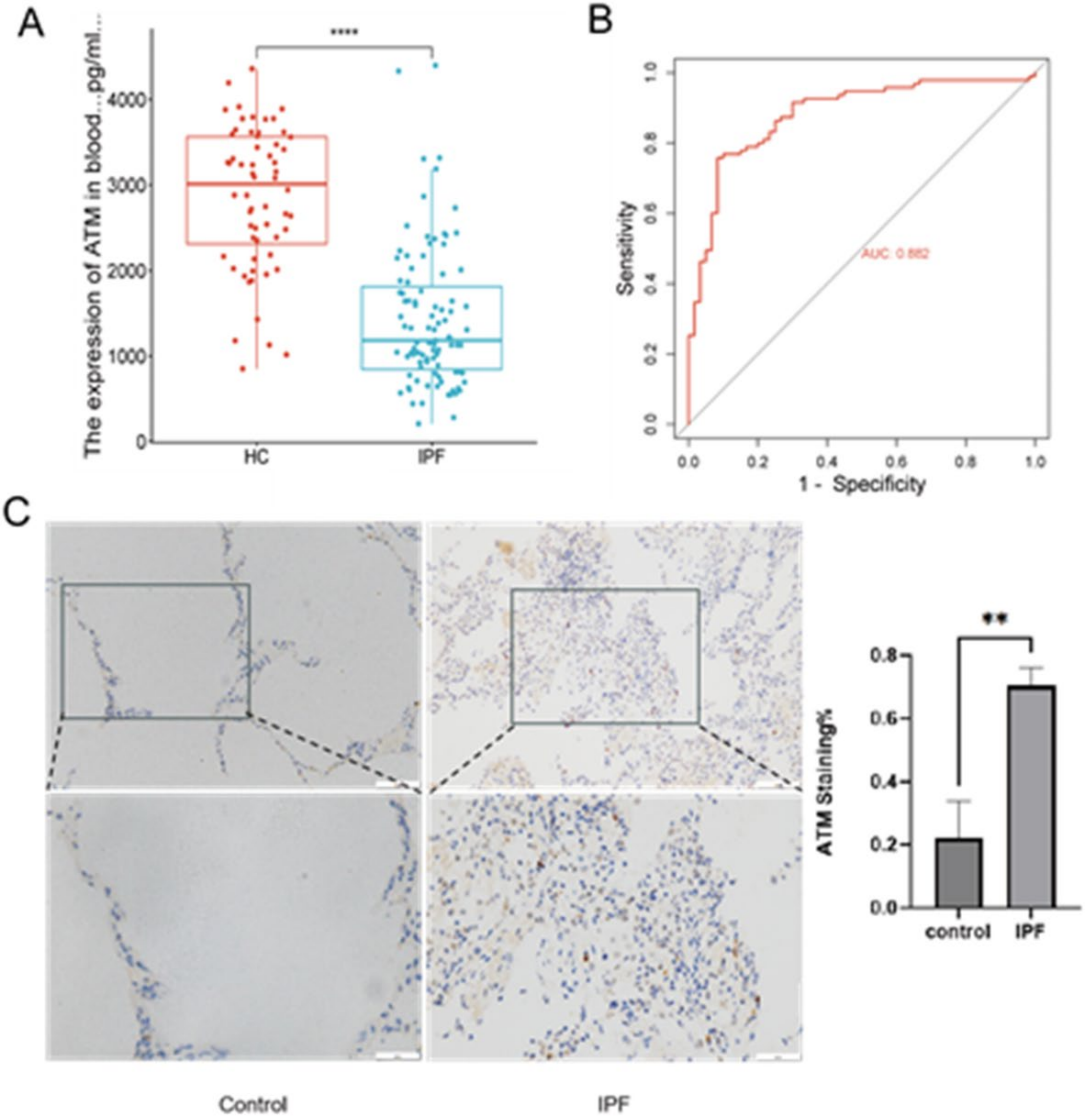
Comparison of ATM expression in the serum and pulmonary tissue between the control and IPF groups: (**A**) Serum ATM concentration in the HC and IPF groups; (**B**) ROC curve of serum ATM levels for IPF diagnosis; (**C**) ATM expression levels in the pulmonary tissues of IPF and controls were detected using IHC. **p < 0.01, ****p < 0.0001.

The serum ATM concentrations in patients with IPF were considerably lower than those in HC (median 3156.24 pg/mL vs. 1262.91 pg/mL, *P* < 0.0001; Fig. 6A). The serum ATM concentrations performed well in IPF diagnosis, with an AUC of 0.882 (Fig. 6B).

### Verification of ATM expression in the pulmonary tissue by IHC

IHC analysis clearly showed distinct pathological changes in the alveolar structure of the IPF group. The alveolar septa thickened remarkably, causing a significant narrowing of the alveolar lumen. Meanwhile, a large number of inflammatory cells infiltrated and obvious focal fibrosis was visible. In contrast, the alveolar structure in the HC group was normal, without these pathological features. IHC results revealed that the cells expressing ATM were preferentially localized in the vicinity of actively fibrotic lesions, including fibrotic foci and the areas surrounding regenerative epithelial cells. Compared with the control group, the expression level of ATM in the nucleus of the IPF group was significantly increased(Fig. 6C).

### Immune infiltration in datasets

**Fig. 7:**
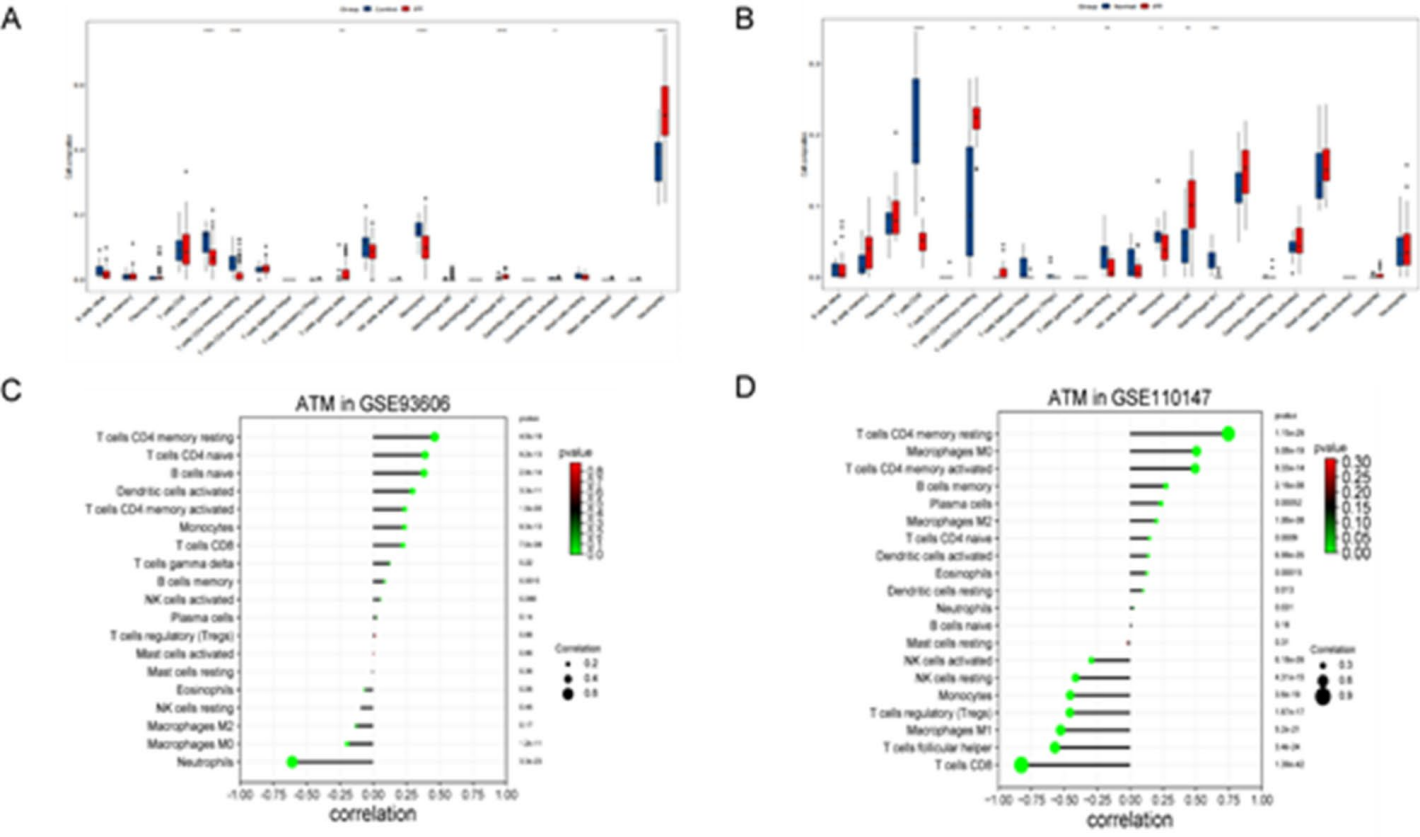
Immunoinfiltration analysis: (**A**) The differences in the immune microenvironment in the serum of patients with IPF and HC in GSE93606; (**B**) The differences in the immune microenvironment in the pulmonary tissue between patients with IPF and controls in GSE110147; (**C**) The relevance between ATM and immune cells in GSE93606; (**D**) The relevance between ATM and immune cells in GSE110147. *p < 0.05, **p < 0.01, ***p < 0.001, ****p < 0.0001.

Figure 7A shows that in the GSE93606 dataset, the infiltration levels of CD4 naïve T cells, CD4 memory resting T cells, monocytes, and resting dendritic cells in the blood of the HC group were higher than those of the IPF group. In contrast, the levels of gamma-delta T cells, M2 macrophages, and neutrophils in the blood of the HC group were lower than those of the IPF group. Figure 7B shows that in the GSE110147 dataset, the infiltration levels of CD8 naïve T cells, follicular helper T cells, Tregs, resting natural killer (NK) cells, monocytes, and M1 macrophages in the pulmonary tissue of the HC group were higher than those of the IPF group. The levels of CD4 memory resting T cells, activated CD4 memory T cells, and M0 macrophages in the pulmonary tissue of the HC group were lower than those of the IPF group.

### Correlation analysis between biomarker and immune cells

Figure 7C shows that in the GSE93606 dataset, ATM in peripheral blood was positively correlated with memory B cells (*r* = 0.088, *P* < 0.01), naïve B cells (*r* = 0.382, *P* < 0.0001), activated dendritic cells (*r* = 0.295, *P* < 0.0001), dendritic cells (*r* = 0.295, *P* < 0.01), monocytes (*r* = 0.235, *P* < 0.0001), activated memory CD4 T cells (*r* = 0.236, *P* < 0.0001), resting memory CD4 T cells (*r* = 0.462, *P* < 0.0001), naïve CD4 T cells (*r* = 0.389, *P* < 0.0001), and CD8 T cells (*r* = 0.223, *P* < 0.0001). On the contrary, it was negatively correlated with neutrophils (*r*=-0.608,*P* < 0.0001) and M0 macrophages (*r*=-0.196, *P* < 0.0001). Figure 7D shows that in the GSE110147 dataset, ATM in pulmonary tissue was positively correlated with M0 macrophages (*r* = 0.745, *P* < 0.0001), resting memory CD4 T cells (*r* = 0.504, *P* < 0.0001), activated memory CD4 T cells (*r* = 0.493, *P* < 0.0001), memory B cells (*r* = 0.271, *P* < 0.0001), plasma cells (*r* = 0.235, *P* < 0.0001), M2 macrophages (*r* = 0.199, *P* < 0.0001), naïve CD4 T cells (*r* = 0.493, *P* < 0.0001), activated dendritic cells (*r* = 0.137, *P* < 0.0001), eosinophils (*r* = 0.127, *P* < 0.001), resting dendritic cells (*r* = 0.099, *P* < 0.05), and neutrophils (*r* = 0.028, *P* < 0.05). However, it was negatively correlated with activated NK cells (*r*=-0.294, *P* < 0.0001), resting NK cells (*r*=-0.414, *P* < 0.0001), monocytes (*r*=-0.455, *P* < 0.0001), M1 macrophages (*r*=-0.527, *P* < 0.0001), Tregs (*r*=-0.457, *P* < 0.0001), M1 macrophages (*r*=-0.527, *P* < 0.0001), follicular helper T cells (*r*=-0.571, *P* < 0.0001), and CD8 T cells (*r*=-0.826, *P* < 0.0001). Overall, ATM was interrelated with immune cells in peripheral blood and pulmonary tissue.

## Discussion

IPF is a chronic age-related disease of undefined pathogenesis, which could lead to a decline in quality of life and early death^1^. Pirfenidone and nintedanib have been approved for the treatment of IPF, which can slow down functional decline and disease progression; however, their effects are limited, and they are associated with tolerance issues^35^. Because of the highly variable and unpredictable clinical progression of IPF^36^, more effective methods for early diagnosis and more effective therapeutic targets are necessary.

Recently, some reports have shown a close relationship between ferroptosis and the development of IPF, but the co-expression of ferroptosis-associated genes in the peripheral blood and pulmonary tissue has not been studied. Therefore, in this study, we identified the co-expressed hub ferroptosis-related gene in the peripheral blood and pulmonary tissue, and validated it using human samples. In our study, the co-expressed hub gene (ATM) related to ferroptosis in the peripheral blood and pulmonary tissues of patients with IPF and HC was detected through data analysis. Peripheral blood and pulmonary tissues were collected from patients with IPF and healthy individuals between 2021 and 2024. The expression levels of ATM were validated by ELISA in serum and by IHC in pulmonary tissues. The variation in immune cells in peripheral blood and pulmonary tissue between patients with IPF and controls in the datasets was estimated. Additionally, the correlations between ATM and immune-infiltrating cells were examined.

ELISA tests revealed that the concentration of ATM in the peripheral blood of patients with IPF was significantly lower than that in the HC group. IHC analysis revealed that the expression level of ATM in the nuclei of the pulmonary tissue of IPF patients was remarkably higher than that in the control group. The AUC value of ATM in peripheral blood was 0.882. The above results were consistent with the dataset results. Meanwhile, multiple studies have suggested that ATM is associated with ferroptosis. ATM may be a good biomarker of ferroptosis after myocardial infarction^37^. ATM is a ferroptosis-related hub gene in obstructive sleep apnea syndrome^38^. ATM protein kinase is a major regulator of double-stranded DNA break signaling and stress responses^39^. ATM kinase, a critical indicator of the reaction to DNA damage, is a key upstream regulator of ferroptosis^40,41^. Mechanistically, ATM kinase phosphorylates the ferritin autophagy receptor NCOA4 and promotes the interaction of NCOA4-FTH1, in order to drive ferritin autophagy-mediated iron mobilisation and exacerbate lipid peroxidation. Deoxynivalenol promotes NCOA4-mediated ferritin autophagy by ATM-NCOA4, subsequently inducing hepatic ferroptosis^42^. The expression of ATM protein is elevated in the pulmonary tissues of mice with pulmonary fibrosis^43^. Inhibiting the activity of ATM protein reduces the expression levels of ECM proteins (such as fibronectin and collagen type I α1 chain) and α -smooth muscle actin in fibroblasts induced by TGF-β^44^. Previous studies have shown that once ATM is activated, it phosphorylates P53 to regulate the expression of P21^45,46^. This process promotes the production of extracellular matrix (ECM) and cellular senescence, thus driving the progression of fibrosis^47,48^. ATM exacerbates the process of fibrosis by promoting the transformation of fibroblasts into myofibroblasts^44,49^. ATM promotes the progression of EMT^50^. In conclusion, ATM may be a good biomarker for ferroptosis in IPF. Notably, the expression of ATM in serum and tissues was completely opposite. We consider that the increased expression of ATM in pulmonary tissue is mainly due to the following reasons: In the pulmonary tissue of patients with IPF, there is continuous oxidative stress and inflammatory response, which leads to damages such as DNA double-strand breaks^51^. This requires the activation of the ATM signaling pathway and an increase in the level of ATM protein. During the onset and progression of IPF, the activation and vigorous proliferation of numerous fibroblasts strongly drive the upregulation of ATM expression. In our view, the reasons for the decrease in ATM protein in the serum of patients with IPF are as follows: a large amount of ATM is recruited to the pulmonary tissue and exerts its function, resulting in a decrease in its distribution in the serum. In patients with IPF, factors such as proteases produced by inflammation may increase the degradation of ATM in the serum^52^.

Since immune responses result in the progression of IPF, the significant distinctions in the immune microenvironment of serum and pulmonary tissue between patients with IPF and controls were found by analyzing immune cell infiltration. The levels of gamma delta T cells, M2 macrophages, and neutrophils in the blood of the control group were lower than those of the IPF group. These results are consistent with previous findings^53^. In the pulmonary tissue of the control group, naïve CD8 T cells, follicular helper T cells, resting NK cells, monocytes, and M1 macrophages had higher infiltration levels compared to the IPF group. On the contrary, in the pulmonary tissue of the control group, resting memory CD4 T cells, activated memory CD4 T cells, and M0 macrophages had lower infiltration levels compared to the IPF group. The results are consistent with Zhang’s findings^54^. It was found that in the GSE93606 dataset, ATM correlated positively with memory B cells, naïve B cells, activated dendritic cells, monocytes, activated memory CD4 T cells, resting memory CD4 T cells, naïve CD4 T cells, and CD8 T cells, whereas it correlated negatively with neutrophils and M0 macrophages. In the GSE110147 dataset, ATM in the pulmonary tissue was positively correlated with resting memory CD4 T cells, M0 macrophages, activated memory CD4 T cells, memory B cells, plasma cells, M2 macrophages, naïve CD4 T cells, activated dendritic cells, eosinophils, resting dendritic cells, and neutrophils. However, activated NK cells, resting NK cells, monocytes, Tregs, M1 macrophages, follicular helper T cells, and CD8 T cells were negatively correlated with ATM. The absence of ATM in CD4 cells leads to premature aging of T cells, thereby promoting HIV infection^55^. The positive correlation between ATM expression and T cells like CD4 + cells and CD8 T cells in serum and pulmonary tissue may be attributed to the crucial role of the ATM protein in the development of the protein κ chain of the T-cell receptor. Specifically, ATM is capable of identifying and mending double-strand breaks during V(D)J recombination^51,56^. Blocking ATM-related DNA damage in T cells could prevent T cell aging mediated by tumor-associated factors and Treg cells in vitro, and enhance antitumor immunity and the effectiveness of immunotherapy in vivo in the context of adoptive transfer T cell therapy^57^. The negative correlation between ATM expression and neutrophils in serum is mainly attributed to the fact that ATM inhibits the NF-κB pathway, thereby reducing the production of neutrophil chemotactic factors such as IL-8^58^. ATM plays a crucial role in the rearrangement of immunoglobulin genes. Abnormal ATM function can impede the normal development process of B cells, resulting in a reduction in the number of mature B cells and thus exerting a negative impact on the immune function of the organism^59^. ATM function deficiency in human B cells induces the secretion of receptor activator of nuclear factor κB ligand and IL-6 in osteoclasts^60^. ATM is crucial in regulating dendritic cell maturation. When ATM is defective, the maturation of dendritic cells is hindered^61^. ATM regulates crucial processes, such as the release of cytotoxic granules in NK cells, directly influencing their ability to eliminate target cells and profoundly affecting the efficiency and effectiveness of immune responses^62^. ATM also plays a crucial role in transforming macrophages into a pro-inflammatory phenotype^63^. ROS regulates M1 macrophage polarisation through ATM and cell cycle checkpoint kinase 2 (Chk2), and the two kinases have a pivotal role in the DNA damage response signalling route^64^. Through comprehensive genomic and transcriptomic analyses of patients with non-small cell lung cancer receiving anti-PD-(L)1 therapy, it was found that the survival rate of patients with ATM mutations was significantly higher than that of patients without ATM mutations^65^. Inhibiting the activity of ATM protein can effectively reverse the EMT process, thus alleviating tumor progression^66^. In summary, since there is a significant correlation between ATM and immune cells in patients with IPF, ATM can serve as a promising target for immunotherapy, opening up new avenues for the development of more effective treatment strategies for this challenging disease.

## Limitations

There are some limitations in this study. Firstly, the research adopted a retrospective analysis method based on clinical data, which inevitably introduced potential selection biases and confounding variables during the research process. Secondly, the number of samples used to verify pulmonary tissues is relatively limited, which, to some extent, restricts the universality of the research conclusions. Therefore, a larger sample size is required to further confirm our research findings. Finally, we preliminarily only verified the expression levels of ATM in human peripheral blood and pulmonary tissues through ELISA and IHC staining, and have not delved deeply into its underlying mechanisms. Thus, it is necessary to conduct further research to comprehensively reveal the underlying molecular mechanisms, so as to gain a deeper understanding of the role of ATM in the process of IPF.

## Conclusion

The important findings could be summarized as follows: By biological information analysis, it was found that ATM was the hub gene related to ferroptosis that co-expressed in the serum and pulmonary tissue of patients with IPF; ELISA verification revealed that the concentration of ATM in the serum of patients with IPF significantly decreased; IHC verification revealed that the expression level of ATM in pulmonary tissue of patients with IPF was significantly increased; there was a significant correlation between ATM and immune-infiltrating cells in serum and pulmonary tissue. In conclusion, ATM can serve as a potential ferroptosis related biomarker for the diagnosis and treatment monitoring of IPF disease, as well as a potential target for immunotherapy.

## Data Availability

The datasets generated during and/or analysed during the current study are available in the Gene Expression Omnibus database, https://www.ncbi.nlm.nih.gov/geo/，GSE93606 and GSE110147.
